# Spider Web DNA: A New Spin on Noninvasive Genetics of Predator and Prey

**DOI:** 10.1371/journal.pone.0142503

**Published:** 2015-11-25

**Authors:** Charles C. Y. Xu, Ivy J. Yen, Dean Bowman, Cameron R. Turner

**Affiliations:** 1 Department of Biological Sciences, University of Notre Dame, Notre Dame, Indiana, United States of America; 2 Potawatomi Zoo, South Bend, Indiana, United States of America; Scientific Research Centre, Slovenian Academy of Sciences and Arts, SLOVENIA

## Abstract

Noninvasive genetic sampling enables biomonitoring without the need to directly observe or disturb target organisms. This paper describes a novel and promising source of noninvasive spider and insect DNA from spider webs. Using black widow spiders (*Latrodectus* spp.) fed with house crickets (*Acheta domesticus*), we successfully extracted, amplified, and sequenced mitochondrial DNA from spider web samples that identified both spider and prey to species. Detectability of spider DNA did not differ between assays with amplicon sizes from 135 to 497 base pairs. Spider and prey DNA remained detectable at least 88 days after living organisms were no longer present on the web. Spider web DNA as a proof-of-concept may open doors to other practical applications in conservation research, pest management, biogeography studies, and biodiversity assessments.

## Introduction

As dominant predators of arthropod communities in natural and agricultural ecosystems, spiders can be important ecological indicators that reflect habitat quality and change [1, 2, 3, 4, 5]. Monitoring the species diversity and abundance of spider assemblages facilitates natural resource management [6]. However, spiders are enormously diverse (~ 45,000 described species) and many can be difficult to identify [7]. Morphological identification of spiders relies primarily on differences in copulatory organs [8] and many complications can prevent identification such as the inability to identify juveniles, extreme sexual dimorphism, size differences between life stages, and genital polymorphisms within species [9–11]. In recent years, genetic identification methods such as DNA barcoding, the use of a short and standardized fragment of DNA to identify organisms, have been growing in popularity because of decreasing costs and ease of use [12]. In particular, the use of DNA barcodes for species identity and systematics of spiders has proven successful in multiple studies [9, 13–15]. The most commonly used genetic marker is the cytochrome c oxidase subunit I (COI) mitochondrial gene because of its designation as the standard DNA barcode [16]. Mitochondrial markers are also ideal for detecting low quantity and quality DNA from environmental or gut samples because each cell contains hundreds to thousands of mitochondrial genomes [17] and there is a positive correlation between gene copy number and detection success [18, 19].

Spiders have a great diversity of life histories and various sampling methods are employed in capturing them including beating, vacuum sampling, sweep netting, pitfall traps, and visual searches. Experiments testing the efficacy of traditional spider sampling methods show high variability in diversity and abundance measurements between methods depending on the habitat and time of sampling. [20–22]. Sampling duration is also an important factor as short-term sampling has been found to reduce the number of recorded species by up to 50% [23]. In this paper, we propose a new biomonitoring tool that would complement existing methods: DNA from spider web. While spider web has been found to efficiently collect pollen, fungal spores and agrochemical sprays [24, 25], no study, to our knowledge, has assessed spider web as a potential source of genetic material. We hypothesized that spider web could be a source of noninvasive DNA from both the spider that built the web and spider prey.

Although noninvasive genetic sampling is most common for vertebrates, it has been successfully applied to arthropod exuviae and frass [26, 27]. Webs are an abundant and easily collected spider secretion that may not only provide spider DNA, but may also function as natural biodiversity samplers that contain environmental DNA (eDNA) from captured prey and other local organisms. This idea parallels recent molecular studies using mosquitos, ticks, leeches, and carrion flies to sample local animal biodiversity [28–31]. Previous studies have successfully used mitochondrial DNA markers to detect spider prey from gut contents, but this requires physically capturing and killing spiders [32, 33]. One recent advance in noninvasive spider diet analysis is the amplification of prey DNA from fecal pellets [34], but fecal pellets are small and may be hard to locate, especially in the field. Furthermore, traditional taxonomic identification of spider prey items is time-consuming, subject to human error, and often only accurate at high taxonomic levels [35]. Spider webs may provide a unique noninvasive opportunity to study arthropod communities without the need to directly observe spider or insect.

We tested the spider web DNA concept by extracting, amplifying and sequencing DNA of black widow spiders, *Latrodectus* spp. (Araneae: Theridiidae), and their prey, the house cricket *Acheta domesticus* (Orthoptera: Gryllidae), from black widow spider webs. Because extraorganismal DNA from spider webs is exposed to environmental degradation and possibly only exists in short fragments, we used nested primer sets to test the effect of amplicon size on detection probability.

## Materials and Methods

### Web collection

The black widow spider exhibit at the Potawatomi Zoo in South Bend, Indiana was inhabited by a single female western black widow spider (*Latrodectus hesperus*) before its death on November 19, 2011. The spider was fed 2 medium sized house crickets (*A*. *domesticus*), on a weekly basis by zookeepers. The exhibit measured 40 cm by 40 cm by 40 cm and contained a few twigs, a small piece of wood, and wood shavings lining the floor. 88 days after the death of the spider, a web sample was collected from the exhibit on February 15, 2012, hereafter referred to as “Lhes_zoo”. The duration of inhabitance within the exhibit prior to the sample collection date is unknown. Three new individual enclosures measuring 35 cm by 30 cm by 35 cm were constructed with plywood and acrylic sheeting and installed on a wall in the zookeeper access hallway behind the exhibit. The enclosures were decontaminated with 10% bleach and installed at the Potawatomi Zoo in South Bend, Indiana.

**Fig 1:**
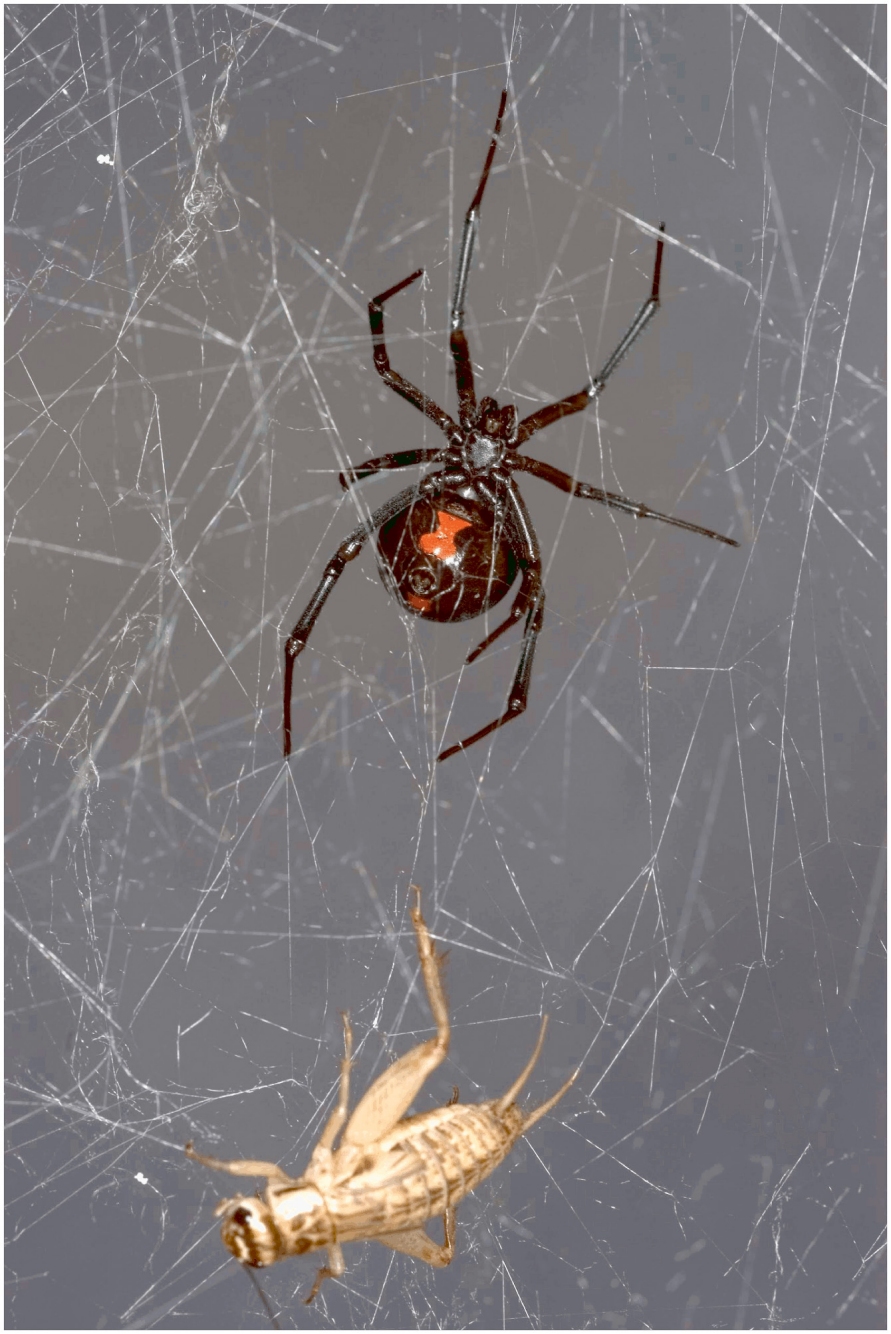
Southern black widow spider (*Latrodectus mactans*) with its prey house cricket (*Acheta domesticus*) trapped in spider web. Image credit Scott Camazine, used with permission.

Three female southern black widow spiders (*Latrodectus mactans*) were purchased from Tarantula Spiders (http://tarantulaspiders.com/). According to the supplier, these spiders were hatched from egg sacs collected in Marion County, Florida, USA and raised on 2–3 housefly maggots (*Musca domestica*) twice per week before delivery to the Potawatomi Zoo. A single live *L*. *mactans* and a bleach-decontaminated branch for web building were placed into each enclosure on April 26, 2012 (Fig 1). After web construction, each spider was fed two medium-sized crickets by dropping them into the web. Web samples were collected from each enclosure 11 days after spider introduction, hereafter referred to as “Lmac_1”, “Lmac_2”, and “Lmac_3”. All web samples were collected by twisting single-use, sterile plastic applicators to spool silk strands. No organism body parts or exuviae were visible in any web samples but cricket parts and spider feces were clearly evident on the floor of the enclosures. Applicator tips were snipped into 1.5-mL microcentrifuge tubes using bleach-decontaminated scissors and stored at -20°C.

### DNA extraction

DNA extractions from web samples were conducted using a modified extraction protocol for shed reptile skins [36]. A negative control without web was also extracted to test for reagent contamination. 800 μL of cell lysis buffer (10 mM Tris, 10 mM EDTA, 2% sodium dodecyl sulfate [SDS], pH 8.0) and 8 μL of proteinase K (20 mg/L) were added to 1.5 mL microcentrifuge tubes containing web samples followed by 10–20 inversions and incubation at 55°C for 4 hours. Upon reaching room temperature, 4 μL of RNase A (10 mg/mL) were added to each sample followed by 20 inversions. Samples were incubated at 37°C for 15 min and then brought back to room temperature. 300 μL of protein precipitation solution (7.5 M ammonium acetate) were added to each sample and vortexed for 20 seconds followed by incubation on ice for 15 min. Samples were then centrifuged at 14,000 rpm for 3 min. Supernatants were transferred to new 2 mL microcentrifuge tubes containing 750 μL of ice cold isopropanol and inverted 50 times before centrifugation at 14,000 rpm for 2 min. All supernatants were drained and 750 μL of 70% ethanol was added to each sample followed by centrifugation at 14,000 rpm for 3 min. All liquids were removed and samples were air dried. DNA pellets were rehydrated using 100 μL of low TE buffer (10 mM Tris, 0.1 mM EDTA).

### Primer design

To detect *Latrodectus* DNA, we designed four nested primer sets based on an alignment of *Latrodectus* COI DNA barcoding sequences obtained from the National Center for Biotechnology Information (NCBI) GenBank database [37, 38]. All four assays included the same forward primer but different reverse primers, producing amplicons of 135 bp, 257 bp, 311 bp, and 497 bp respectively (Table 1). To detect prey eDNA, we designed a set of primers that specifically amplifies 248 bp of the DNA barcoding region of the COI gene in *A*. *domesticus* using sequences obtained from the NCBI GenBank database (Table 1) [39, 40]. GenBank accession numbers of DNA sequences used to design all primers are provided in S1 Table.

**Table 1:** PCR primers designed to amplify the cytochrome c oxidase subunit I (COI) gene of target species.

Primer name	Sequence (5’-3’)	Size (bp)	Amplicon (bp)	Target taxon
Lat_COI_F1	GAATTAGGGCAACCGGGAAG	20	-	*Latrodectus* spp.
Lat_COI_R1	AGGAACTAATCAATTTCCAAACCCC	25	135	*Latrodectus* spp.
Lat_COI_R2	CCAGCTCCAACCCCAACC	18	257	*Latrodectus* spp.
Lat_COI_R3	ACAGAACTTCCTCTATGTCCTTCCAA	26	311	*Latrodectus* spp.
Lat_COI_R4	GCCCCTGCTAATACAGGTAAT	21	497	*Latrodectus* spp.
Adom_F	TGGTGGATTCGGAAATTGAT	20	-	*A*. *domesticus*
Adom_R	CCCGCAAGAACAGGTAAAGA	25	248	*A*. *domesticus*

All *Latrodectus* spp. primer sets are nested and use the same forward primer.

### DNA amplification

All DNA samples were amplified in polymerase chain reactions (PCR) of 20 μL containing 13.28 μL of ddH_2_O, 2 μL of 5 PRIME^®^ 10x Taq Buffer advanced, 2 μL of 5 PRIME^®^ Magnesium Solution at 25 mM, 0.4 μL of dNTPs at 2.5 mM, 0.12 μL of 5 PRIME^®^ Taq DNA polymerase at 5 U/μL, 0.6 μL of forward and reverse primers at 10 μM, and 1.0 μL of DNA template using Eppendorf Mastercycler^®^ pro thermocyclers. Cycling conditions were as follows: 94°C/5 min, 55X (94°C/20 s, 54.4°C/35 s, 72°C/30 s), 72°C/7 m, 4°C/hold. Each *Latrodectus* spp. primer set was used to amplify all DNA samples with 10 technical replicates to measure detection probability for different amplicon sizes. All web DNA samples were amplified with 2 technical replicates using the *A*. *domesticus* primer set. One negative control reaction with ddH_2_O instead of DNA template was included on every PCR plate to test for contamination. Gel electrophoresis was conducted using 5 μL of PCR product mixed with 3 μL of loading dye and 10 μL of ddH_2_O. Multiple wells were loaded with 5 μL of 100 bp ladder (Promega) on each gel. Technical replicates showing amplicons of the expected size were pooled and purified using ExoSAP-IT (Affymetrix). Bi-directional Sanger sequencing using ABI BigDye chemistry (Life Technologies) was conducted on an ABI 3730xl 96-capillary sequencer by the University of Notre Dame Genomics Core Facility. Sequencing chromatograms were primer- and quality-trimmed in Sequencher (ver. 5.0; Gene Codes Corp.). Internal ambiguous base calls were denoted as “N”. BLASTn searches of the NCBI GenBank database [41] and Barcode of Life database (BOLD) Identification System (IDS) COI searches of Species Level Barcode Records with default settings were used for taxonomic identification of COI barcode sequences. For each query sequence, the resulting match with the highest percent identity (90–100%) was accepted for taxonomic identification. Accession and IDS numbers of top NCBI and BOLD matches, respectively, are provided in S2 Table.

## Results

**Fig 2:**
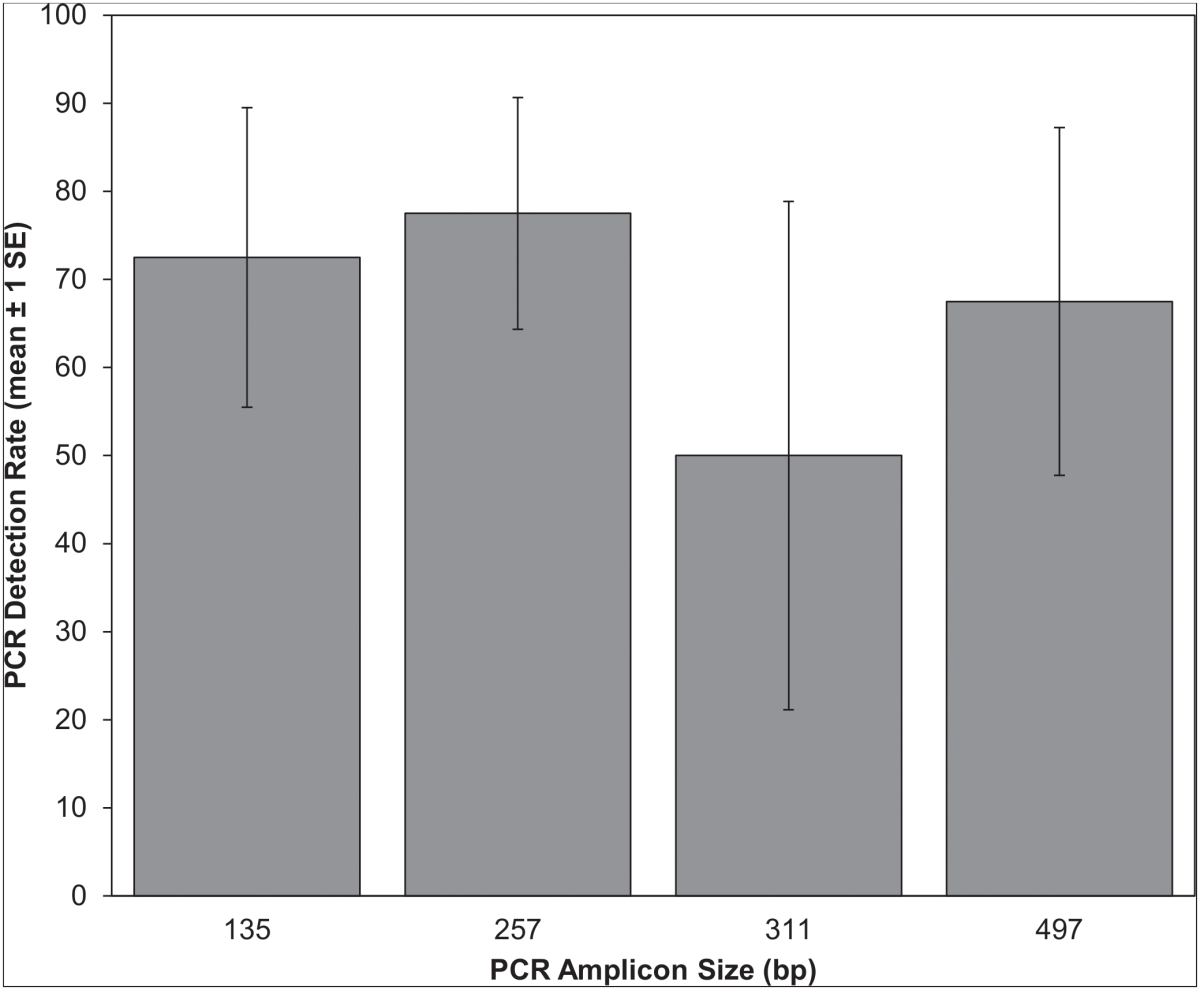
Success of different amplicon sizes in detecting the cytochrome c oxidase subunit I (COI) of *Latrodectus* spp. from web samples. Percent success calculated from number of successful PCRs out of 10 technical replicates. Error bars represent ± 1 standard error.

All extraction and PCR negative controls produced no amplification. Using the nested primer sets, we successfully amplified 135 bp, 257 bp, 311 bp, and 497 bp of *Latrodectus* spp. COI from web DNA samples (Fig 2). With the exception of zero amplification for the 311 bp PCR assay from two samples, 2–10 technical replicates of each PCR assay successfully amplified from all samples. Web DNA sequences obtained from enclosure samples, “Lmac_1”, “Lmac_2”, and “Lmac_3”, were confirmed by NCBI BLAST and BOLD IDS to be *L*. *mactans* and DNA from the zoo exhibit sample, “Lhes_zoo”, was confirmed to be *L*. *hesperus*. Two sequences contained short internal runs of ambiguous base calls that did not prevent taxonomic identification (S2 Table). Amplicon size had no effect on PCR success based on the number of successful PCR replicates (ANOVA, F = 1.941, d.f. = 3, *P* = 0.194). We also successfully amplified 248 bp of *Acheta domesticus* COI from web DNA samples. Both PCR duplicates from all four web samples amplified successfully and all resulting DNA sequences were confirmed by NCBI BLAST and BOLD IDS to be *A*. *domesticus*. The zoo exhibit web sample, “Lhes_zoo”, was collected 88 days after the death and removal of both spider and prey, demonstrating substantial persistence of web DNA. All DNA sequences generated in this study are provided in S2 Table.

## Discussion

The present study represents, to our knowledge, the first demonstration of spider web as a source of noninvasive genetic material. Spider web is an ideal source of noninvasive genetic material for spiders because web can be found and collected without the need to directly observe or capture spiders themselves. Furthermore, unlike most spiders, which are small, mobile, and elusive, webs are relatively large, stationary, and often clearly visible. Spider webs may also remain after spiders move or die, which increases detection probability especially for more elusive species. Webs can also exist in great abundance. For example, web coverage may reach up to more than 50% of land area in agricultural fields [42]. Spider webs have already been utilized by citizen scientists to assess spider biodiversity through visual analysis of web structure [43] and it could be possible to implement similar citizen science initiatives to collect web samples for DNA analysis.

Because black widow spiders are cobweb spiders that generate large three-dimensional cobwebs consisting of sheets dotted with glue droplets [44], they were ideal to use in this experiment. Although spider silk could be considered a form of spider tissue, spider silk fibers are composed of tightly bound β-sheet proteins that exclude water molecules and do not dissolve under the proteinase K treatment of standard DNA extraction protocols [45]. Thus, we hypothesize that most spider web DNA originates either from microscopic pieces of fecal matter, setae, and exuviae adhered to silk strands or directly from the silk gland exudate, which may contain cells shed from silk glands.

Certain black widow spiders like the species used in this study are common venomous pests [46] and spider web DNA could be a particularly useful surveillance tool. Spider web DNA could also help monitor low density populations and determine invasion fronts of invasive widow spiders such as the brown widow, *Latrodectus geometricus*, in southern California and the Australian redback, *Latrodectus hasseltii*, in New Zealand and Japan [47, 48]. Besides pest and invasive species, many spiders like the katipo, *Latrodectus katipo*, are threatened or endangered [49]. The geographic range and abundance of thousands more spider species are unknown but may be declining. Spider web DNA could be particularly useful in rapidly providing occurrence and genetic diversity data for these rare species of concern. As a noninvasive biomonitoring method, spider web DNA could be used for conservation and taxonomy without sacrificing organisms that are already threatened by human disturbance. The collection and genetic analysis of spider webs could also serve spider biogeography studies, which require large-scale sampling across wide geographic ranges [38].

Our proof-of-concept experiment used spider web from indoor enclosures where DNA-degrading conditions such as heat, moisture, and light were likely reduced relative to field conditions. Many spider taxa, including *Latrodectus*, build webs in protected spaces [50], but further testing of field-collected spider web from more species and habitats is needed to evaluate the generality of our findings. Nevertheless, this first demonstration suggests a promising approach for arthropod monitoring. The ability to target particular species could be useful in monitoring low density populations of pest, invasive, or endangered insects. Future work using massively parallel sequencing on spider web eDNA could reveal entire assemblages of arthropods in a cost-effective manner, especially with the rapid advancement and decreasing costs of such technologies [51]. This method could be used for diet analysis, which would be especially useful in assessing the importance of riparian spiders as links between aquatic and terrestrial food webs [52]. In some environments such as temperate forests, approximately 40% of arthropod biomass is annually consumed by spiders [53]. Although spider predation cannot be concluded from the mere presence of DNA on spider webs and it is unlikely that individual web samples will consistently yield DNA of the full diversity of spider prey, detection of insect eDNA from spider web does at least indicate local proximity. Spider web eDNA may complement traditional assessment methods of local arthropod biodiversity and potentially reveal previously undiscovered biodiversity through improved sensitivity and sampling effort [54]. Such information regarding species diversity is critically important in conservation planning and environmental impact assessments [55, 56]. However, it is crucial to note that DNA barcoding is most valuable in combination with the taxonomic expertise necessary to provide species identities. The successful use of spider web DNA relies heavily on having well-annotated DNA sequences available such as those found in BOLD. Without quality reference databases, species-level identification of rare, endangered, or invasive species is difficult. Generation of new sequence data along with proper annotation will improve the usefulness and efficacy of this new tool.

In conclusion, we provide a proof-of-concept that noninvasive DNA of a spider and its prey can be extracted from spider web and be used for species identification. Spider web DNA appears to be a promising tool with wide applications in biomonitoring, biogeography and biodiversity assessments of spiders and their prey, especially if combined with the power of massively parallel sequencing [57].

## Supporting Information

S1 Table GenBank accession numbers of DNA sequences used to design primers targeting spiders (*L*. *hesperus*, *L*. *mactans*) and prey (*A*. *domesticus*). (XLSX)

S2 Table Cytochrome c oxidase subunit I sequences of spiders (*L*. *hesperus*, *L*. *mactans*) and prey (*A*. *domesticus*) generated from spider web DNA. (XLSX)
